# Clinical Implications of Digenic Inheritance and Epistasis in Primary Immunodeficiency Disorders

**DOI:** 10.3389/fimmu.2017.01965

**Published:** 2018-01-26

**Authors:** Rohan Ameratunga, See-Tarn Woon, Vanessa L. Bryant, Richard Steele, Charlotte Slade, Euphemia Yee Leung, Klaus Lehnert

**Affiliations:** ^1^Department of Virology and Immunology, Auckland City Hospital, Auckland, New Zealand; ^2^Department of Clinical Immunology, Auckland City Hospital, Auckland, New Zealand; ^3^Department of Immunology, Walter and Eliza Hall Institute of Medical Research, Parkville, VIC, Australia; ^4^Department of Medical Biology, University of Melbourne, Parkville, VIC, Australia; ^5^Department of Allergy and Clinical Immunology, Royal Melbourne Hospital, Parkville, VIC, Australia; ^6^Auckland Cancer Society Research Centre, University of Auckland, Auckland, New Zealand; ^7^School of Biological Sciences, University of Auckland, Auckland, New Zealand

**Keywords:** common variable immunodeficiency disorders, epistasis, digenic mutation, primary immunodeficiency disorder, monogenic syndromes

## Abstract

The existence of epistasis in humans was first predicted by Bateson in 1909. Epistasis describes the non-linear, synergistic interaction of two or more genetic loci, which can substantially modify disease severity or result in entirely new phenotypes. The concept has remained controversial in human genetics because of the lack of well-characterized examples. In humans, it is only possible to demonstrate epistasis if two or more genes are mutated. In most cases of epistasis, the mutated gene products are likely to be constituents of the same physiological pathway leading to severe disruption of a cellular function such as antibody production. We have recently described a digenic family, who carry mutations of *TNFRSF13B*/TACI as well as *TCF3* genes. Both genes lie in tandem along the immunoglobulin isotype switching and secretion pathway. We have shown they interact in an epistatic way causing severe immunodeficiency and autoimmunity in the digenic proband. With the advent of next generation sequencing, it is likely other families with digenic inheritance will be identified. Since digenic inheritance does not always cause epistasis, we propose an epistasis index which may help quantify the effects of the two mutations. We also discuss the clinical implications of digenic inheritance and epistasis in humans with primary immunodeficiency disorders.

## History of Epistasis

Epistasis was a term coined by Bateson in 1909 (1). Based on a series of experiments, Bateson predicted there would be interactions between two or more genetic loci to produce novel phenotypes. Bateson and Punnett undertook experiments on the genetics of flower color in peas and comb morphology in roosters. When white pea flowers were crossed, all of the F1 hybrid was purple, a new phenotype. When the purple F1 hybrids were crossed, the result was unexpected: a ratio of 9:7 purple to white colored flowers. This result could not be explained by Mendelian genetics (2).

Bateson and Punnett described a similar phenomenon in the comb morphology of roosters. They speculated there must be at least two genes, which were interacting in order to explain their observations. Bateson’s predictions were made long before the structure of DNA was elucidated and underlying biochemical pathways were understood. It is now known that there are two enzymes, which lie in tandem along the pathway for the production of the purple anthocyanin pigment (3). Homozygous null mutations of either enzyme result in white colored pea flowers.

**Figure 1:**
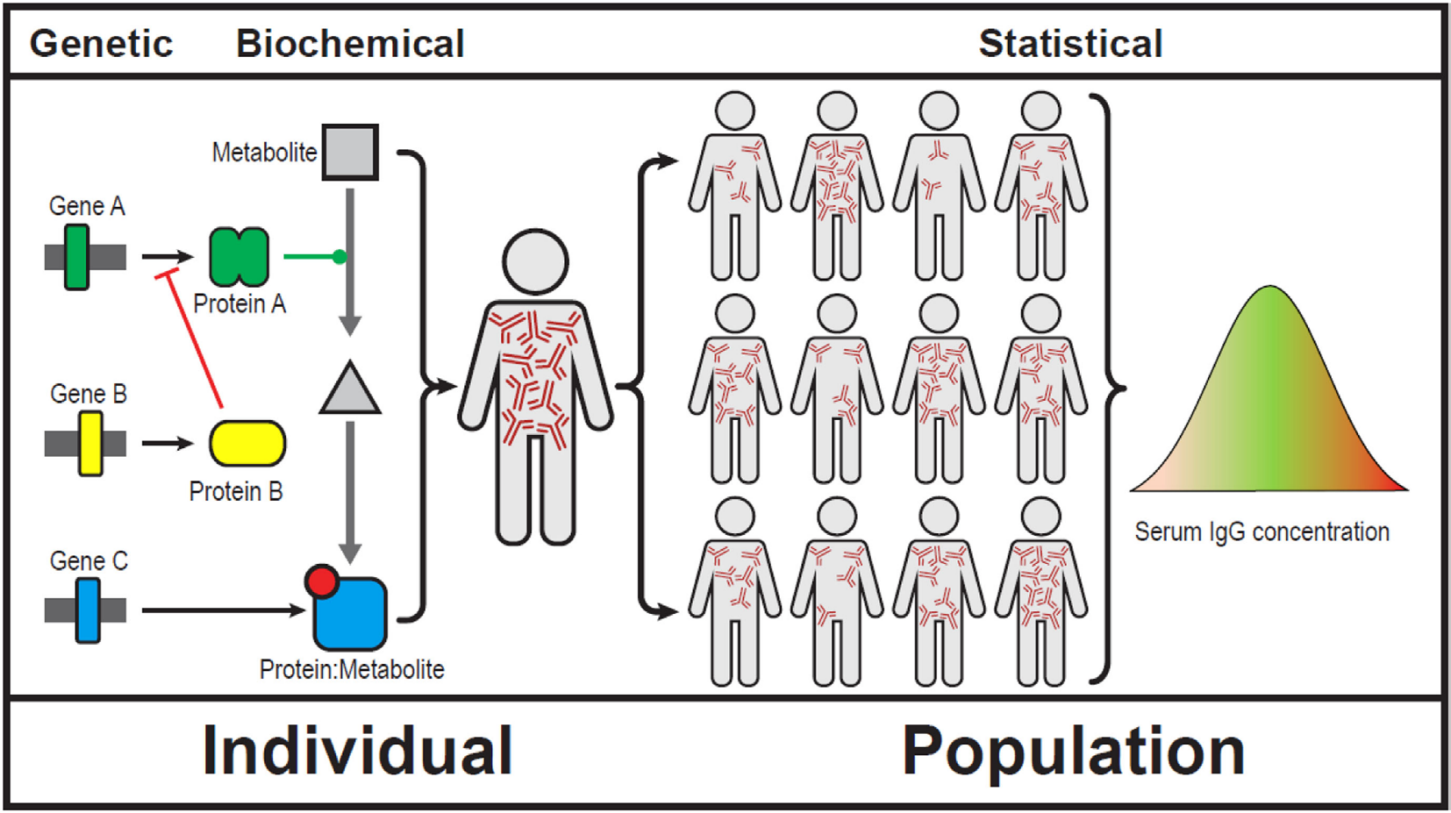
Showing epistasis at a genetic, biochemical, clinical, and population level (5). We use serum immunoglobulins to show gene–gene interactions can result in broad array of serum IgG levels. Note that IgG levels do not follow a Gaussian distribution in human populations.

Bateson and Punnett’s observations could not be explained at the time and the concept of epistasis became controversial. Sir Ronald Fisher was famously quoted “no epistasis on my watch.” Over time epistasis became a statistical concept (4). It is much easier to demonstrate interactions of multiple genes in a population than in a single individual (Figure 1).

## Studies in Non-Mammalian Organisms and Laboratory Animals

Epistasis plays an important role in evolution. Multiple mutations may confer a survival advantage in a rapidly changing environment and therefore enhance reproductive fitness. A dramatic clinical example is the swift evolution of bacterial multidrug antibiotic resistance in intensive care units. In the face of extensive antibiotic usage, bacteria are under intense selection pressure to quickly evolve drug resistance to maintain reproductive fitness. Strains rapidly developing multiple antibiotic resistance from the epistatic interactions of two or more mutations (or acquiring resistance elements horizontally) are at a survival advantage (6).

In yeast, combinations of two different genetic variants can result in a phenotype that is different from each individual strain, i.e., qualitative epistasis (7). Subsequent work with iRNA screens to knock down gene function in *Caenorhabditis elegans* and *Drosophila melanogaster* has revealed that genetic networks are likely to be similarly affected in eukaryotic systems (8).

Epistasis can also be explored in laboratory animals (9). Single gene mutations can arise either spontaneously or may be induced by a variety of techniques including gene targeting or CRISPR-Cas9 gene editing technology. Cross-breeding allows the exploration of the phenotypic effects of digenic (or multigenic) disorders.

There are, however, caveats to such an approach. There may be substantial phenotypic differences between humans and mice carrying the same mutations (10). This could complicate interpretation of data in mice. We have also discussed problems utilizing CRISPR-Cas9 gene edited mice to replicate human genetic disease (11). Apart from the technical difficulties creating these mice, there is the potential for undetected off-target effects. This could compromise the interpretation of data.

## Studies in Humans

Because of the lack of well-characterized examples, epistasis is not a commonly used term in human clinical genetics. In general, epistatic interactions occur along a single biochemical pathway where mutations can aggravate (negative epistasis), ameliorate (positive epistasis), or produce an entirely new phenotype. We suggest that positive and negative epistasis are grouped as quantitative epistasis while gene–gene interactions resulting in a new phenotype are termed qualitative epistasis. This simplified classification makes no presumptions on how digenic disorders are inherited or how the genes interact.

In the case of quantitative epistasis, there should be a clinical method for measuring the severity of the phenotype such as a disease-specific severity score. If clinical epistasis is to be demonstrated in an individual with digenic inheritance, the disease severity score should be much worse (or much better) compared with family members bearing only one mutation. The results could be quantified as an epistasis index (EI) as described below. If the biochemical basis of the clinical disorder is known, epistasis can potentially be quantified and confirmed *in vitro*. In an ideal model, clinical (phenotypic) epistasis should mirror relevant *in vitro* functional studies (biochemical epistasis) in family members (Table 1).

**Table 1:** Requirements for confirming epistasis in humans.

Investigating epistasis in humans
**Genetic epistasis**
There must be more than one mutated gene in a single kindred The family should display a range of possible permutations and combinations of the two mutations, i.e., a genetically informative kindred There should be at least one member of the family with wild-type alleles for both mutations

**Clinical epistasis**
Each mutation should have a clear clinical phenotype There must a be method of assessing the clinical severity of the disorder, i.e., a clinical disease-specific severity score The clinical severity score should mirror the pattern of the mutations

**Biochemical epistasis**
The mutations should affect molecules known to interact or participate in the same pathway There should be a readily available assay for measuring the effects of these proteins on the biochemical pathway The *in vitro* tests should show disruption of the pathways These biochemical pathways should be *in vitro* correlates for the disease phenotype, i.e., the biochemical defect should be congruent with the clinical phenotype The severity of the *in vitro* test scores should reflect pattern of the mutations

**Clinical, genetic, and biochemical abnormalities must be congruent**
There should be close correlation between the clinical severity score and the disruption of the *in vitro* assays

*Having an informative family carrying two or more mutations is required for demonstrating genetic, biochemical, and clinical epistasis in humans. Animal models may or may not be successful given the caveats described in the text*.

Unlike individuals bearing a single gene defect, we suggest that epistatic effects of digenic disorders can only be fully evaluated in an informative family (Table 1). Each of the mutations must have a demonstrable phenotype, even if mild. In order to demonstrate epistasis with confidence, both mutations should be present in at least one family member (the proband) and there must be individuals carrying only one of the mutations in the kindred (Table 1). Individuals with neither mutation serve as the wild-type control for the family.

## Digenic Inheritance in Human Autoimmunity

Recently, a consanguineous Kuwaiti family was described with mutations of both the *LRBA* and *NEIL3* genes (12). Three children bearing both mutations died at a young age from severe autoimmunity. The authors speculated that interaction of the two mutations may have contributed to the severe clinical manifestations. Interestingly, an unrelated Kuwaiti woman with the same homozygous *NEIL3* deficiency had no clinical manifestations of autoimmunity. The frequency of *NEIL3* mutations in the Kuwaiti population is approximately 2%. Because the three children had died, the authors did not have the opportunity to undertake detailed clinical or quantitative functional studies outlined in Table 1. Both mutations in this digenic disorder may have had a similar effect of predisposing to autoimmunity (category #5 of digenic disorders in Table 3).

## Epistasis in Primary Immunodeficiency

Primary immunodeficiency disorders (PIDs) are rare genetic defects of immunity predisposing to infections, autoimmunity, allergies, and malignancy. To date, over 300 monogenic defects causing PIDs have been described (16). There are many clinical advantages in identifying the genetic basis of these conditions (Table 2).

**Table 2:** Advantages of molecular analysis for primary immunodeficiency disorders (PIDs).

Diagnosis of PID
Distinguishing genetic from acquired disorders
Confirming the clinical diagnosis
Identifying novel presentations of PIDs
Identifying atypical presentations of PIDs
Identifying cases of phenocopy
Urgent diagnosis in infancy where conventional diagnostic tests are unreliable

**Treatment**
Assisting treatment decisions
Gene therapy-identifying those who may benefit from gene-based therapy
Therapies targeting epistatic gene products or their constituent pathway(s)
Specific treatments based on the mutated molecule, e.g., abatacept for *CTLA4* or *LRBA* deficiency

**Prognosis**
Patients with causative genetic defects have a high probability of symptomatic disease
Patients with genetic defects are unlikely to recover spontaneously cf. infections

**Presymptomatic testing**
Where presymptomatic diagnosis (at any age) is not possible with protein-based tests, e.g., *SH2D1A*
Early identification of disorders which present later in childhood, e.g., hereditary angioedema

**Screening**
Cascade screening of at-risk relatives
Population-based screening

**PID prevention**
Prenatal diagnosis chorion villus sampling
Preimplantation genetic diagnosis

**Research**
Characterizing the role of molecules in cellular function
Assisting with the classification of primary immunodeficiency disorders
Identification of new genetic defects including animal models
Drug development targeting the mutated pathway

*The original case descriptions can be found in our review on PID genetic testing. Updated from our previous publications, permitted under Biomed Centrals copyright rules (13, 14)*.

Common variable immunodeficiency disorders (CVID) are the most frequent symptomatic primary immunodeficiencies in humans and can present from infancy to late in life. CVID is characterized by late onset antibody failure (LOAF) leading to immune system failure (ISF). Over the last 5 years, an increasing number of CVID patients have been identified with monogenic disorders (17–20). Most of these patients were identified with the assistance of next generation sequencing (NGS) (21). Once identified, these patients are removed from umbrella diagnosis of CVID and are deemed to have a CVID-like disorder (22, 23).

We have recently shown that a digenic CVID-like disorder resulted from epistatic interactions of two mutated genes: transcription factor 3 (*TCF3*) and *TNFRSF13B*/TACI (21). The proband, who has both mutations is severely affected with multiple infections and she also meets the American College of Rheumatology criteria for systemic lupus erythematosus.

In this family, virtually all combinations of mutations were present allowing us to quantify the severity of disorder in order to investigate epistasis (Table 1; Figure 3). The clinical severity, measured by the clinical score for CVID, closely matched the pattern of the mutations (24). *In vitro* antibody production studies, which are the ultimate correlates of LOAF/ISF, mirrored the clinical phenotype of each family member (Figure 3). *In vitro* antibody production was most severely impaired in the digenic proband and milder defects were identified in other family members carrying a single mutation.

A consanguineous family was recently described, where the proband has mutations of the *IFNAR1* and *IFNGR2* genes. The patient was susceptible to mycobacterial, bacterial, and viral infections. There were no other siblings in the family. Given the autosomal recessive nature of the two mutations, both parents were carriers and were asymptomatic. In this case, it is likely both mutations had a similar function (category #5, Table 3) (25).

**Table 3:** Patterns of digenic inheritance, expanded from Gazzo et al. (15).

(1) Directly interacting genes/proteins (2) Indirectly interacting genes/proteins (3) Common pathway (4) Co-expression (RNA) (5) Similar function of genes/proteins (6) No obvious link in genes/proteins, i.e., different pathways (7) Sequence variations which do not produce a discernible phenotype (8) Sequence variations which do not alter protein expression, e.g., non-synonymous variants

*We suggest future examples of digenic inheritance are classified according to this scheme*.

## Patterns of Digenic Inheritance and EI

In a recent review, the authors suggested digenic disorders could be categorized according to the likely interactions of genes (Table 3) (15). An extended version is shown in Table 3 and it might be predicted that not all cases of digenic inheritance result in epistasis. Mutations of genes in unrelated pathways may not interact (category #6). In contrast, epistasis may occur in directly interacting genes as well as those functioning at different positions along the same pathway (categories #1 and #3). In order to further quantify this, we propose an EI. We suggest the following equation to identify non-linear effects of two or more mutations. Higher numbers indicate more severe disease (e.g., clinical score when computing CVID severity).

$$\text{Epistasis index}=\frac{\text{Digenic score}}{M1+M2},$$

where *M*1 and *M*2 refer to scores of individuals bearing each mutation. When a lower number (e.g., *in vitro* antibody production) indicates greater severity, the equation can be inverted:

$$\text{Epistasis index}=\frac{M1+M2}{\text{Digenic score}}.$$

In either case, an EI value >1 indicates a synergistic effect consistent with negative epistasis. In the case of positive epistasis, the scores will be <1 indicating mitigation of effect. It is possible the EI is >1 for some *in vitro* parameters but not others. If there is no epistasis, the EI will be 1 indicating additive effects of the mutations.

We suggest computing epistasis indices independently, using the clinical disease severity score and the most informative biochemical marker to demonstrate epistasis, in this case *in vitro* antibody production. The EI can be applied to quantitative epistasis but not qualitative epistasis, where there is a completely different phenotype, such as a change in the color of pea flowers in F1 and F2 hybrids. As stated above, the EI cannot be computed from the digenic individual alone, in the absence of family members bearing each of the mutations.

In the case of the family described above, the calculated results are as follows:

Clinical EI (Based on the clinical score, Figure 3B) 34/(13 + 3) = 2.13
Biochemical EI (*In vitro* IgG production, nanogram per milliliter: Figure 3C) (749 + 647)/138 = 10.1

It should be noted that sequence variations in the absence of a phenotype (category #7, Table 3) do not necessarily indicate digenic or oligogenic inheritance. These sequences may be occurring in deeply intronic regions or they may be non-synonymous variants, with no effect on protein translation. In such situations, there may be no phenotype associated with these variants, making epistasis unlikely.

## Discussion: Clinical Implications of Digenic Inheritance and Epistasis

The most important aspect of digenic inheritance is its clinical recognition. Failure to recognize digenic disorders could have catastrophic consequences for patients. It was previously thought mutations of *TNFRSF13B*/TACI caused CVID (26). More recently, *TNFRSF13B*/TACI mutations are perceived to have only mild effects on the phenotype (27) and mostly function as a risk allele for CVID and related disorders. Had this family been advised to undertake implantation genetic diagnosis (PGD)/chorion villus sampling (CVS) or amniocentesis based on the *TNFRS13B*/TACI mutation alone, serious consequences could have resulted from this error.

Even if recognized, digenic disorders will complicate diagnostic testing in the case of PGD. At this time, it seems unlikely both mutations could be reliably identified by single cell PCR for PGD. There is high probability of allele drop out, leading to a false-negative result. Given access to greater amounts of tissue and therefore DNA, CVS, and amniocentesis are more likely to reliably identify both mutations. We have indicated priority should be given to identifying the more damaging mutation (*TCF3*) for PGD or CVS/amniocentesis (13, 14).

Similarly, the more deleterious *TCF3* mutation is likely to determine long-term prognosis and should be utilized when making therapeutic decisions such as administration of SCIG/IVIG. As seen in this and similar CVID-like disorders, it is likely most individuals bearing causative mutations will become symptomatic at some stage in their lives. In this family, the individuals heterozygous for the *TNFRSF13B*/TACI mutation alone are unlikely to require immunoglobulin replacement in the absence of the concomitant *TCF3* mutation.

**Figure 2:**
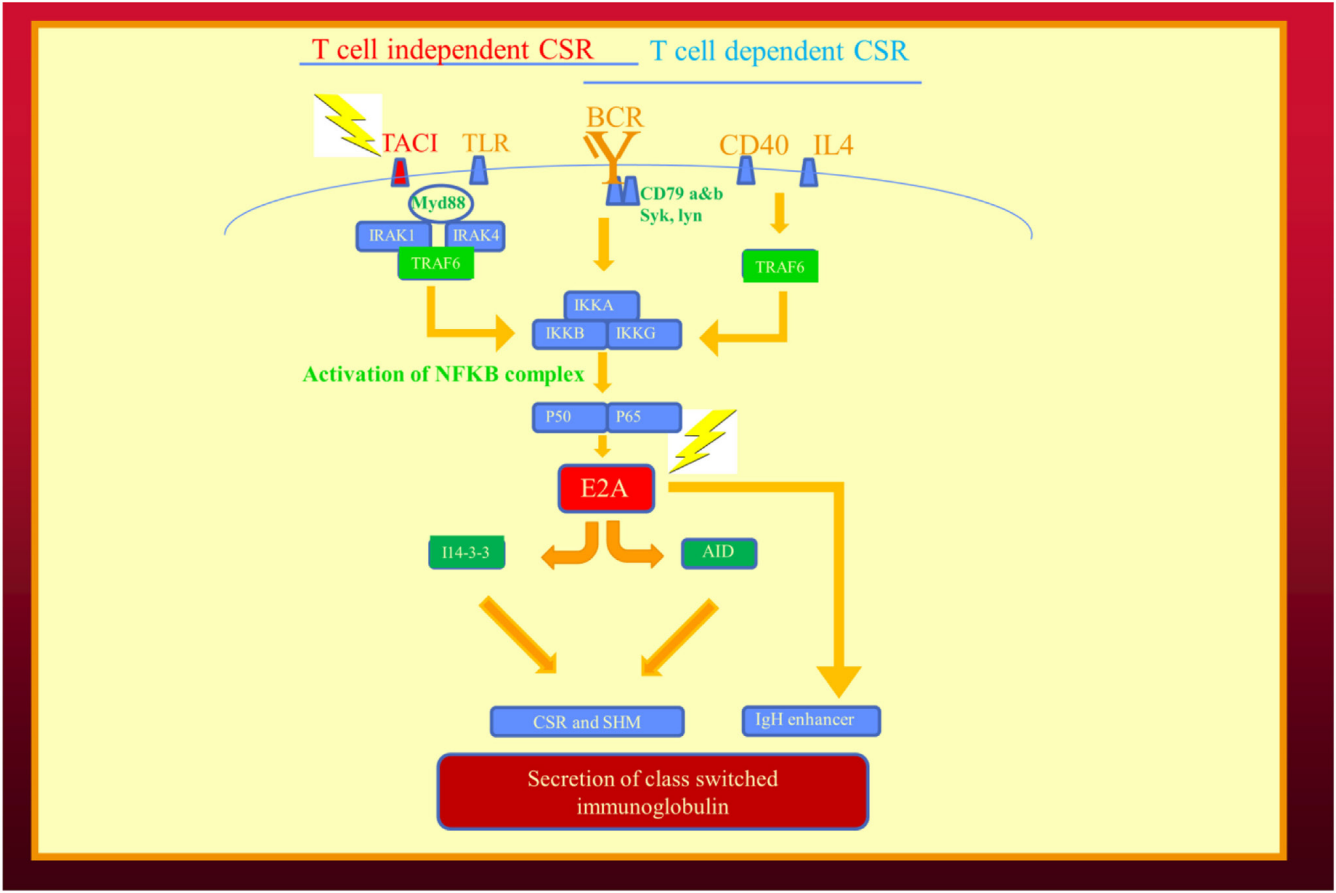
Epistatic interaction of mutated genes in the immunoglobulin isotype switching and secretion pathways from our recent publication describing human epistasis (21). TACI plays a critical role in T cell independent isotype class switching while E2A/(TCF3) affects both pathways. I14-3-3 is a scaffolding protein for activation induced cystidine deaminase. Mutations depicted by lightening. BCR, B cell receptor; CSR, immunoglobulin class switch recombination; SHM, somatic hypermutation; TLR, toll-like receptors.

As in the example of flower color in peas and immunoglobulin production our family, it is likely future examples of digenic inheritance leading to epistasis will involve molecules along the same biochemical pathway (Table 3). In our family, one molecule was a cell surface receptor (*TNFRSF13B*/TACI) and the second more significant mutation (*TCF3*) was in a transcription factor (Figure 2). The *TCF3* mutation can be considered a “hub” in the epistatic interaction of the two pathways (9). Being a transcription factor, *TCF3* influences the expression and function of multiple molecules: activation-induced cystidine deaminase, I14-3-3, and immunoglobulin production (Figure 2) (28). Given its broad range of effects, it is not surprising the *TCF3* is the critical mutation in this digenic family and the reason we consider it the epistatic hub. In future cases of epistasis, one molecule is likely to be a transcription factor or a regulatory molecule in a signaling pathway.

**Figure 3:**
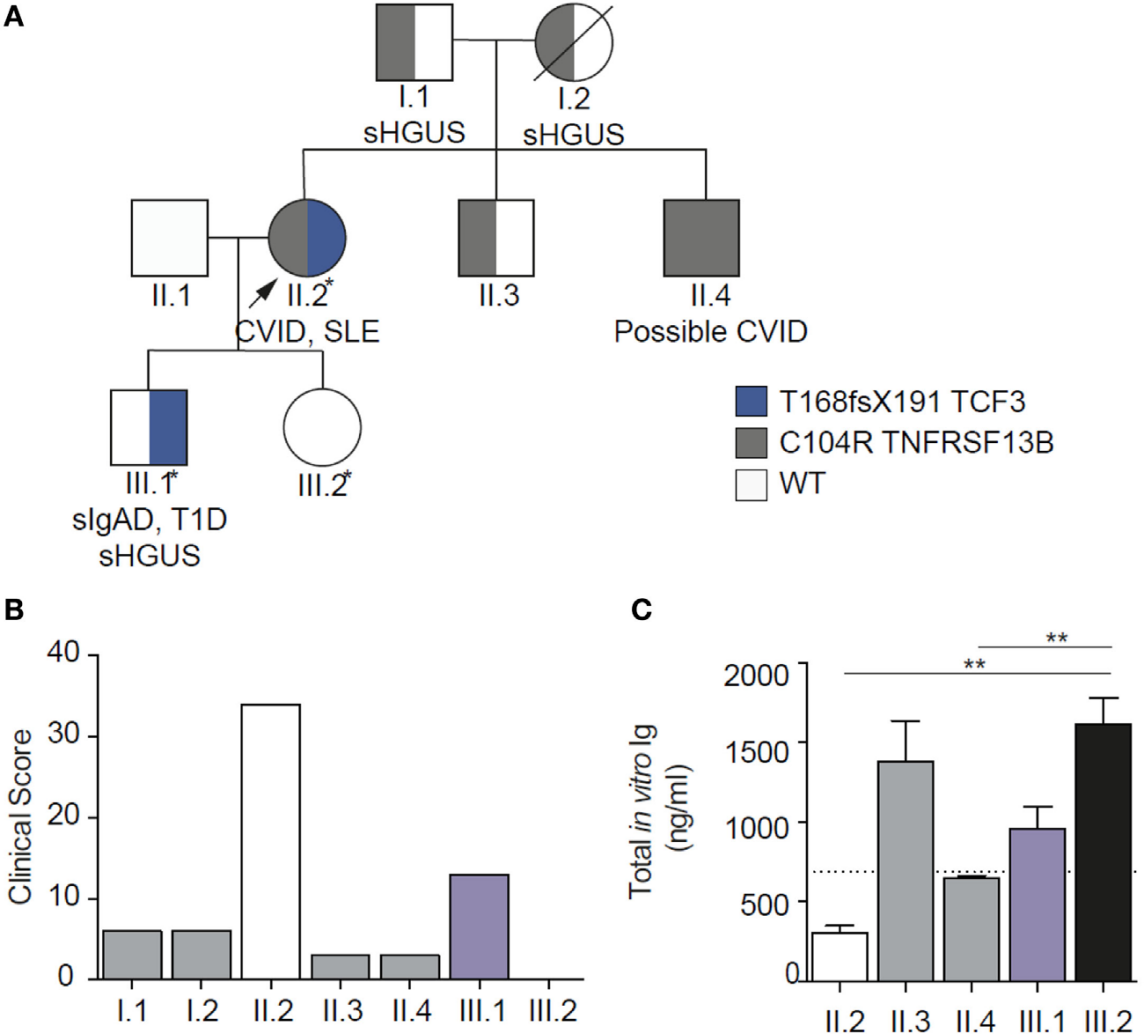
Family pedigree showing genetic, biochemical, and clinical epistasis. **(A)**. Showing the digenic kindred, with inheritance of the two mutations. **(B)** Clinical epistasis shown with the clinical score. The proband carrying both mutations is much more severely affected than the sum of her symptomatic son [bearing the transcription factor 3 (TCF3) mutation] or any of the other individuals heterozygous for TACI mutation. Note that the clinical score for the unaffected daughter (III.2) is 0. **(C)** *In vitro* IgG production through the T cell independent pathway, showing severely impaired *in vitro* IgG production in the proband carrying both mutations. Diagram modified under the creative commons license (21).

Our findings in this family also justify the segregation of genes predisposing to CVID from those causing a CVID-like disorder (22, 23). We have included genes predisposing to CVID (*TNFRSF13B*/TACI, *TNFRSF13C*/BAFFR, *TWEAK*, and *MSH5*) in the Ameratunga et al. CVID diagnostic criteria primarily for patients enrolled in clinical research studies (23). As discussed previously, the prevalence of mutations in these predisposing genes is much higher than the lifetime estimates of CVID in the same population (29). We and others have also shown these mutations do not segregate with symptomatic family members (30). This is convincing evidence that these mutations cannot be causative in the absence of other mutated genes. In this digenic family, those individuals bearing the more severe mutation (*TCF3*) are classified as having CVID-like disorders, while those with *TNFRSF13B*/TACI mutation remain within the broad spectrum of CVID (31).

Our observation has shed new light on the role of *TNFRSF13B*/TACI mutations. In this family, it appears that the *TNSRSF13B*/TACI mutation has a modifying effect on the *TCF3* mutation. The phenotype of the *TCF3* mutation is exacerbated (negative epistasis) by the presence of the *TNFRSF13B*/TACI mutation in the proband (category #3, Table 3). We have previously advised against the routine sequencing of genes predisposing to CVID such as *TNFRSF13B*/TACI, *TNFRSF13C*/BAFFR, *TWEAK*, and *MSH5* (22). Given the mild influence on phenotype, their role has not been clear. It now appears they may be playing an epistatic role, at least in some patients. Given their newly discovered role, there may be an argument for sequencing these genes, particularly if a more significant genetic defect has already been identified in a family.

This is the first example of digenic inheritance resulting in a CVID-like disorder as a result of epistasis (21). Both mutations are required for the complete phenotypic expression of the disorder in this proband. We have classified this digenic condition as a CVID-like disorder as the genetic basis is understood (22). Such digenic disorders will require a new classification of PIDs as neither mutation alone can cause the full phenotype on its own in this family (21). Similarly, they will require two OMIM gene annotations when identified. A recently curated digenic database will be very helpful in understanding these complex disorders (15). It will be important to document if clinical and biochemical epistasis has been confirmed in an informative digenic family.

A recent retrospective study suggested that up to 5% of patients undergoing NGS had a digenic disorder (32). Other examples of digenic inheritance leading to PIDs are likely to be discovered in the future. As in the family we have described, the initial clue indicating the presence of digenic inheritance will emerge from family studies showing lack of co-segregation between the pattern of symptoms and the inheritance of the initial candidate mutation. It will be important to ensure the phenotypic features of each family member can be satisfactorily explained by the functional effects of the initially identified mutation. If there is a discrepancy in the segregation analysis, there may be another unidentified mutation, as seen in our family. It is likely the first mutation will be identified by Sanger sequencing of a candidate gene (33) and the second mutation will be obtained by NGS-based gene discovery techniques.

In the absence of consanguinity, causal mutations are more likely inherited in an autosomal dominant manner, or arise *de novo* as seen in the New Zealand family described in our publications (21). This crucial information will inform the curation strategy in NGS, and the NGS data should be used to determine consanguinity in case this knowledge is not known to, or available from the family. The curation strategies for NGS will thus depend on the frequency of consanguinity in the population being surveyed. It will also be critical to understand the frequencies of candidate variants in population(s) representing the proband’s ethnic population. There is a serious risk of assigning disease significance to a common polymorphism in an under-surveyed ethnic group.

If none of the identified candidate genes are known to cause an immunodeficiency in a patient with PID, this will complicate evaluation of the digenic disorder. The role of each gene may have to be determined separately prior to attributing the epistatic effects of both mutations to the phenotype of the proband. There should be no discrepancy when the phenotypic and laboratory effects of each mutation are correlated with the clinical features of each member of the kindred. Within the limits discussed above, reproduction of the orthologous mutation in laboratory animals may support the existence of epistatic interactions between the two genes.

A computer program predicting epistasis *in silico* will greatly advance the investigation of digenic disorders and might assist in cases where the effects of genes are not completely understood (15). Such programs may attempt to quantify epistatic effects from network perturbation scores, score all pairwise contributions to known pathways, or simply identify pairs of genes acting in the same pathway. Particularly exciting are recent studies that compute tolerance to loss-of-function mutations for each gene from large-scale surveys of human genetic variation (34, 35). These scores may be combined with the approaches above to refine candidate pairs and prioritize follow-up experiments. For example, observing fewer loss-of-function mutations in *TCF3* than statistically expected in 60,000 healthy individuals strongly suggests that humans are intolerant to this type of mutation in *TCF3*. In contrast, the same metric suggests that loss-of-function mutations in *TNFRSF13B*/TACI are better tolerated (27). The global genome sequencing efforts (now exceeding variants from 250,000 chromosomes) should allow a similar estimation of intolerance to loss-of-function mutations affecting gene pairs.

Investigation of this family has given us insight into previously unanswered questions in human genetics, which were first raised over a century ago. Apart from being a well-characterized example of human epistasis our observation offers a molecular explanation for variable penetrance and expressivity. It has long been speculated that individual genetic variation in modifying genes could be the basis of variable penetrance and expressivity. Other explanations are of course possible including environmental influences and epigenetic changes in methylation patterns leading to altered gene expression (36). Investigation of similar families will not only identify new PID disorders but will fundamentally change our understanding of human heredity.

## Ethics Statement

All studies were approved by Auckland Hospital (3435), NZ Ministry of Health (MEC/06/10/134), and the Walter and Eliza Hall Institute (WEHI) Human Research Ethics Committee (HREC 10/02).

## Author Contributions

RA conceptualized the article and wrote the first draft. S-TW contributed to molecular biology section in non-human models. VB contributed to the figure and text. Performed functional work in the original paper. RS contributed to conceptualization and helped edit the manuscript. CS contributed to original paper through functional assays. EL undertook practical work on the epistasis project and contributed to the text. KL played a major role in the bioinformatics section.

## Conflict of Interest Statement

All authors declare they have no competing interests. All studies were undertaken with the appropriate consent and institutional ethics approvals.
